# A novel, nontoxic iron chelator, super-polyphenol, effectively induces apoptosis in human cancer cell lines

**DOI:** 10.18632/oncotarget.25973

**Published:** 2018-08-28

**Authors:** Toshiaki Ohara, Yasuko Tomono, Xing Boyi, Sun Yingfu, Kazuhiro Omori, Akihiro Matsukawa

**Affiliations:** ^1^ Department of Pathology & Experimental Medicine, Okayama University Graduate School of Medicine, Dentistry and Pharmaceutical Sciences, Okayama, Japan; ^2^ Department of Gastroenterological Surgery, Okayama University Graduate **School** of Medicine, Dentistry and Pharmaceutical Sciences, Okayama, Japan; ^3^ Shigei Medical Research Institute, Okayama, Japan; ^4^ Department of Periodontics and Endodontics, Okayama University Hospital, Okayama, Japan

**Keywords:** iron, chelation, chelator, apoptosis, toxicity

## Abstract

Iron chelation therapy is the main treatment for iron overload disease. Iron chelators were recently reported to be useful for cancer therapy; however, they cause side effects that make them difficult to use in some cancer patients. Thus, a novel oral iron chelator, super-polyphenol (SP), was developed for cancer therapy to decrease the side effects. SP is either water soluble or insoluble, and has different isoforms according to the number of side chains. Of these isoforms, water-soluble SP6 and SP10 appear to be the best candidates, as they have the strongest chelating abilities. In this study, we focused on the usefulness and safety of SP6 and SP10 as anti-cancer drugs, and examined their anti-cancer effects and toxicity. The results showed that SP6 and SP10 inhibited cancer cell proliferation by inducing apoptosis in HCT116, HSC-2, A549, and MCF-7 cancer cells. SP10 also inhibited tumor growth in an HCT116 xenograft model. SP6 and SP10 had no acute toxicities. An intravenous injection test revealed that SP6 and SP10 had better safety profiles than the iron chelator deferoxamine. In conclusion, SP is a novel oral iron chelator with anti-cancer effects and few adverse side effects. This is the first report of SP in the literature.

## INTRODUCTION

Iron is an essential element for all living organisms and plays an important role in critical cellular processes such as energy production and DNA synthesis. Although adequate iron levels are essential for human health, iron overload causes some disorders such as hemochromatosis, which is often referred to as iron overload disease [1]. In addition, iron overload is a common complication in patients receiving blood transfusion as a treatment for conditions such as aplastic anemia and myelodysplastic syndrome [2]. Moreover, iron overload causes carcinogenesis in some organs [3] by causing oxidative damage of DNA by the Fenton reaction [4]. Thus, iron depletion by an iron chelator has been explored as a possible therapeutic intervention in cancer. Some iron chelators have been shown to inhibit cancer cell proliferation, either alone or in combination with other anti-cancer drugs [5–8]. However, iron chelators can cause potentially serious side effects. For example, deferasirox (DFX), an oral iron chelator, has superior iron chelation ability, but causes digestive, liver, and kidney disorders [9]. Deferoxamine (DFO) is an intravenous iron chelator that also has toxic side effects [10]. Decreasing the side effects of iron chelators may improve cancer treatment compliance, thereby improving clinical outcomes.

A novel iron chelator was developed by Dr. Nishida [11] for the reduction of side effects, and was named “super-polyphenol (SP).” SP is either water-soluble or insoluble. Water-soluble SP has few side effects as it is thought to avoid metabolism by cytochrome P450; drug metabolism via the cytochrome P450 system can cause drug interactions that result in drug toxicity [12, 13]. SP has several isoforms according to the number of side chains. Of these, water-soluble SP6 and SP10 are thought to be the best candidates, as they have the strongest chelating abilities. Thus, in this study, we focused on the usefulness and safety of SP6 and SP10 as anti-cancer drugs, and examined their anti-cancer effects and toxicity.

## RESULTS

### SP6 and SP10 chelated ferric ion in a dose-dependent manner

**Figure 1:**
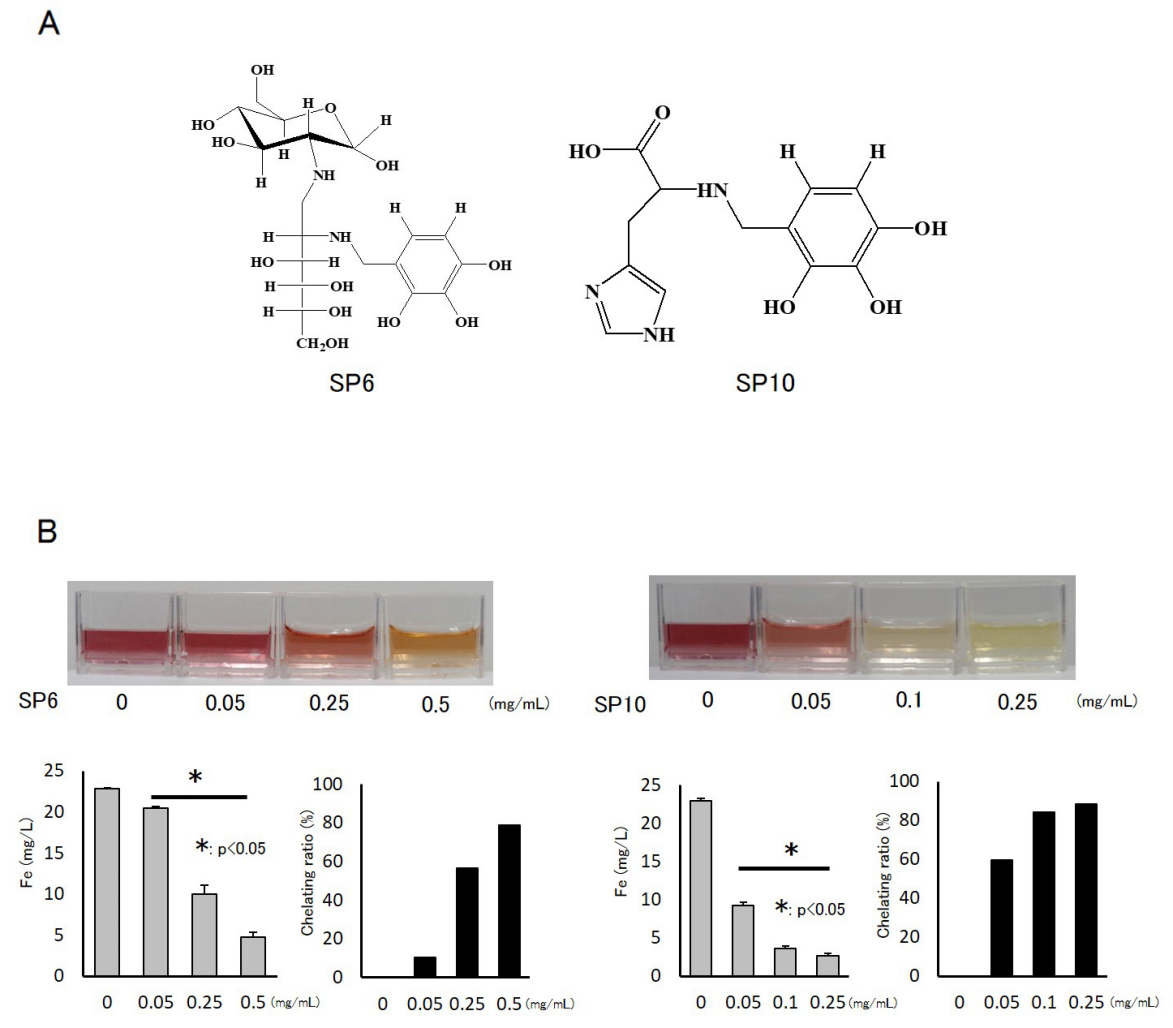
Structure and chelating abilities of SP6 and SP10 **(A)** SP was chemically synthesized from catechol and benzoic acids. SP6 was made by a condensation reaction with glucosamine, and SP10 was made by a condensation reaction with histidine. **(B)** The basic chelating abilities of SP6 and SP10 were examined by standard Fe^3+^ water solution and the sulfosalicylic acid visual colorimetric method. SP6 and SP10 were added to the standard Fe^3+^ solution (22.4 mg/L). The color of the liquid changed from red to yellow according to Fe^3+^ chelation. Data are presented as the mean ± standard deviation (SD) (n=4; ^*^p<0.05).

SP6 was made by a condensation reaction with glucosamine, and SP10 was made by a condensation reaction with histidine (Figure 1A). We examined their basic chelating abilities using standard ferric (Fe^3+^) water solution by the sulfosalicylic acid visual colorimetric method. SP6 and SP10 chelated Fe^3+^ in a dose-dependent manner (Figure 1B). A dose of 0.5 mg/mL SP6 chelated more than 75% of iron, and 0.1 mg/mL SP10 chelated more than 80% of iron. These results indicated that the chelating ability of SP10 was stronger than that of SP6.

### SP6 and SP10 suppressed cancer cell proliferation by inducing apoptosis

**Figure 2:**
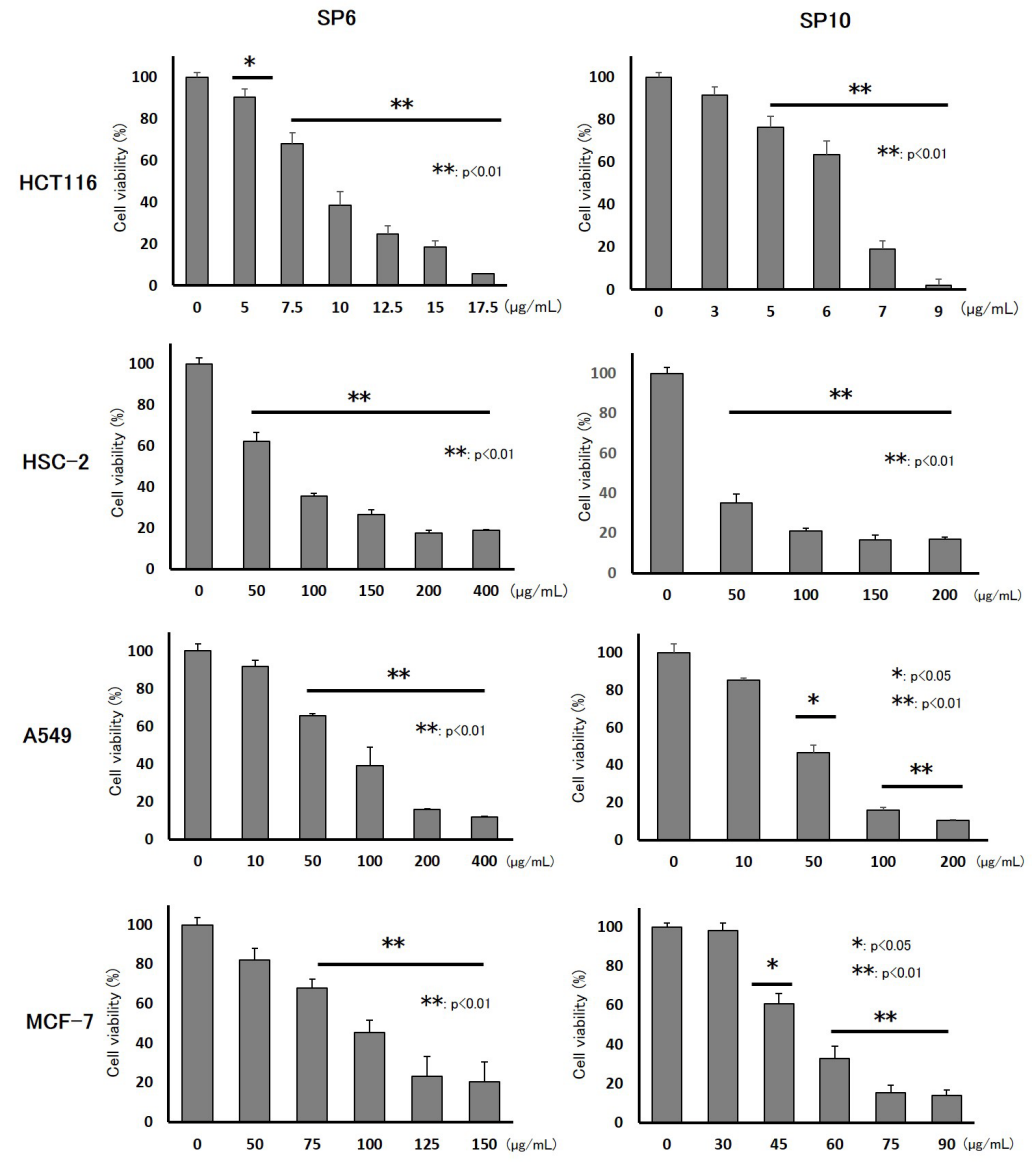
Inhibitory effects of SP6 and SP10 in cancer cells *in vitro* Cultured HCT116, HSC-2, A549, and MCF-7 cells were treated with different concentrations of SP6 and SP10 for 72 h, after which cell viability was evaluated using the XTT assay. Cell viability in the absence of treatment was set at 100%. The results are the means of three independent experiments. Data are presented as the mean ± standard error of the mean (SEM) (n=3; ^*^p<0.05, ^**^p<0.01).

**Figure 3:**
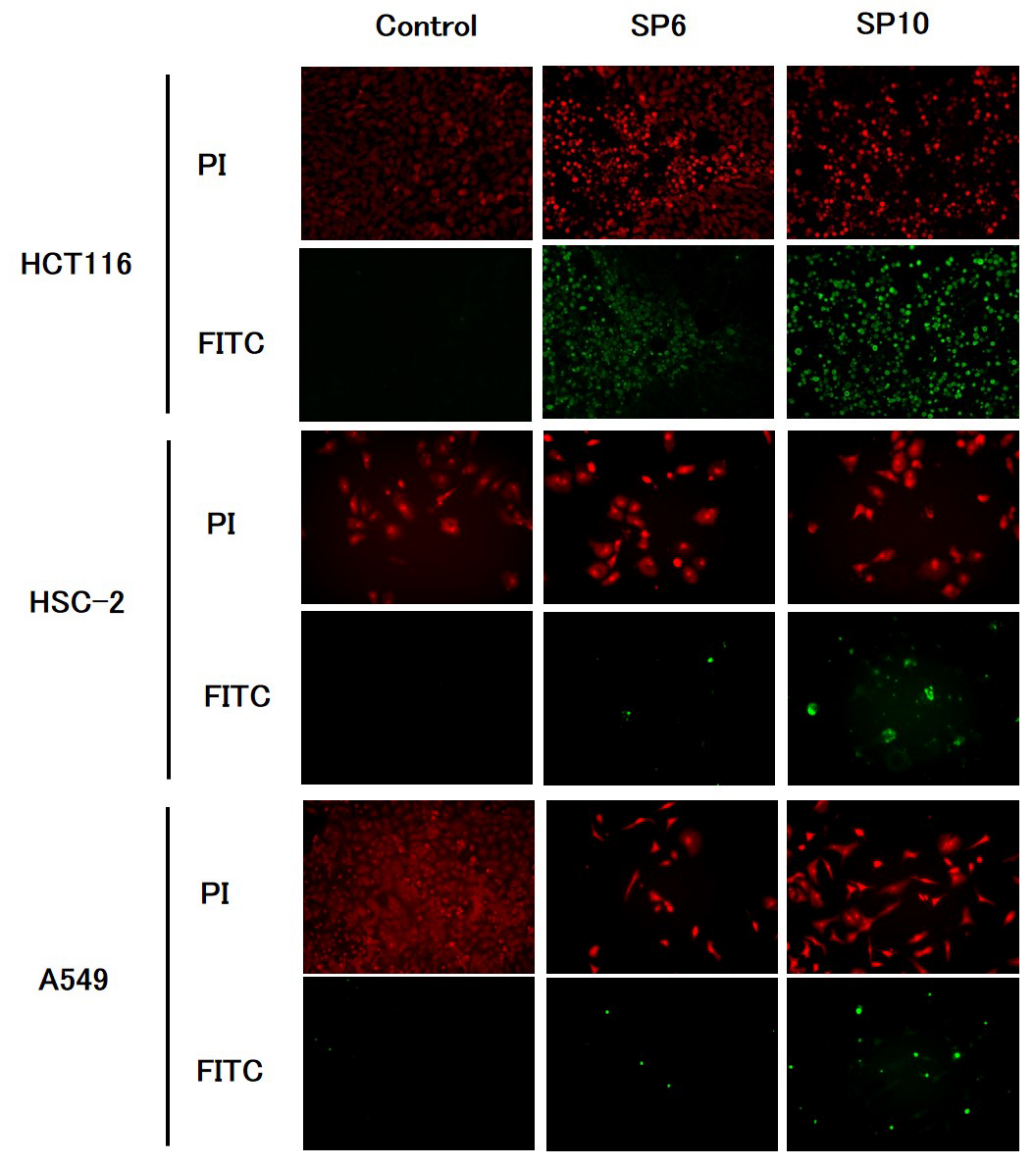
TUNEL staining of cancer cells *in vitro* Cultured HCT116, HSC-2, A549, and MCF-7 cells were treated with different concentrations of SP6 and SP10 for 72 h, and TUNEL staining was performed to detect apoptosis. The concentrations of SP6 used were as follows: HCT116: 10 μg/mL, HSC-2: 100 μg/mL, and A549: 100 μg/mL. The concentrations of SP10 used were as follows: HCT116: 6.5 μg/mL, HSC-2: 50 μg/mL, and A549: 50 μg/mL. PI-stained nuclei were red in color. Apoptotic cells detected as FITC-positive cells were green in color. Apoptotic cells were detected in both the SP6 and SP10 treatment groups.

**Figure 4:**
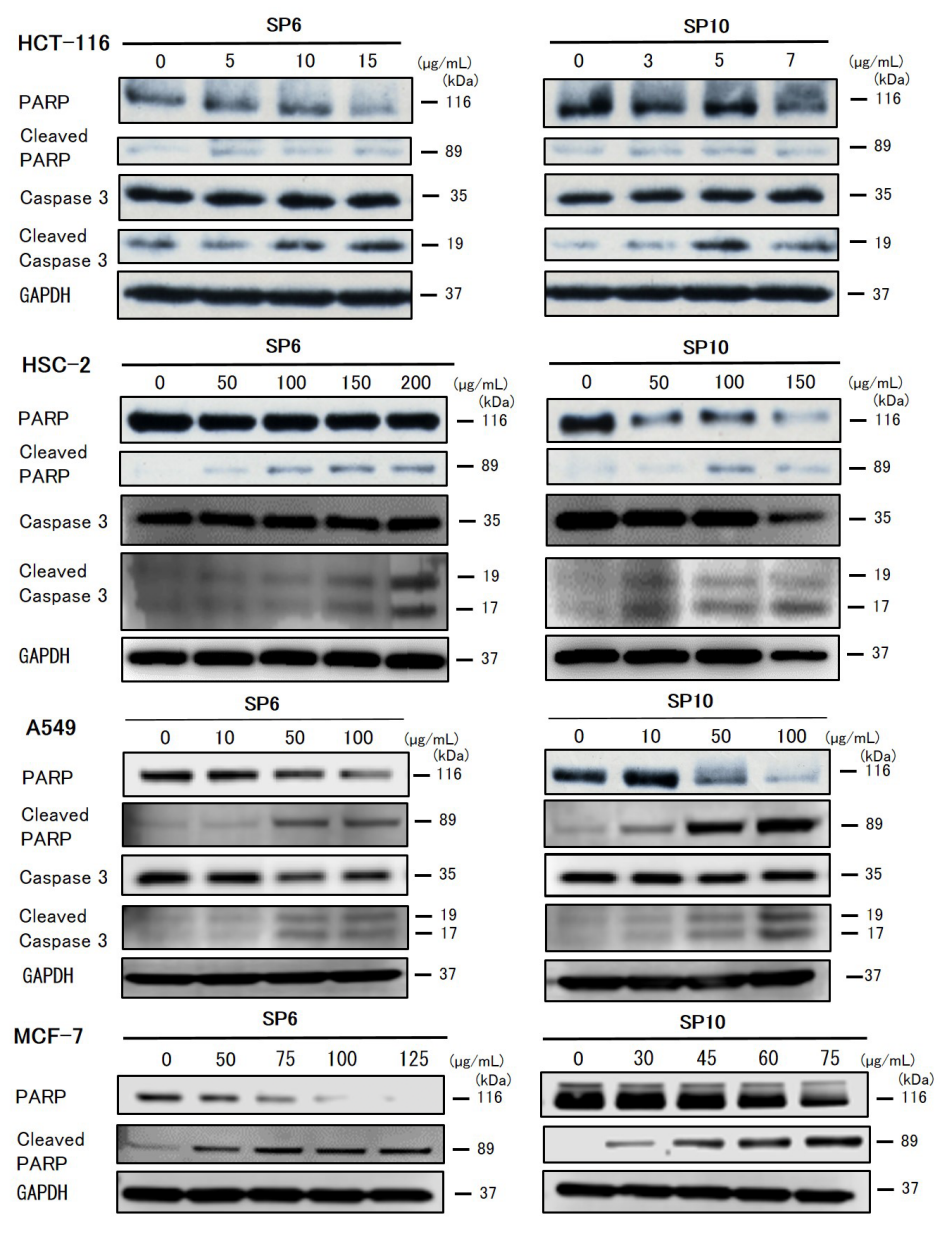
Western blot analysis of apoptosis in cancer cells *in vitro* Cultured HSC-2, A549, and MCF-7 cells were treated with different concentrations of SP6 and SP10 for 72 h. Then the cells were harvested and expression of the indicated proteins was analyzed. SP6 and SP10 induced expression of the apoptotic markers, cleaved caspase 3 and cleaved PARP.

To evaluate the anti-cancer effects of SP6 and SP10, we examined the cell viability of four cancer cell lines using the XTT cell viability assay. SP6 and SP10 suppressed the proliferation of cancer cells in a dose-dependent manner (Figure 2). The IC_50_ value was 9.3–93.2 μg/mL for SP6 and 6.2–51.4 μg/mL for SP10 (Table 1). HCT116 cells were most sensitive to SP6 and SP10. We used the terminal deoxynucleotidyl transferase dUTP Nick-End Labeling (TUNEL) assay to determine the mechanisms underlying their anti-proliferative effects. TUNEL staining revealed apoptotic cells in the SP6 and SP10 treatment groups (Figure 3), which was quantified by Image J software analysis (Supplementary Figure 2A). To confirm the apoptotic effects, we performed western blot analysis (Figure 4). The expression of cleaved poly (ADP-ribose) polymerase (PARP), a DNA repair enzyme, was increased in HCT-116, HSC-2, A549, and MCF-7 cells in a dose-dependent manner. The expression of cleaved caspase 3 was also increased in HCT-116, HSC-2, and A549 cells in a dose-dependent manner. MCF-7 cells are caspase 3-deficient [14]. These results indicated that SP6 and SP10 suppressed the proliferation of cancer cells by inducing apoptosis.

**Table 1:** IC_50_ of SP6 and SP10 in cancer cells

Cell line	IC_50_ (μg/mL)	
	SP6	SP10
HCT116	9.3	6.2
HSC-2	67.7	22.1
A549	90.8	41.4
MCF-7	93.2	51.4

IC_50_ values (μg/mL) of SP6 and SP10 after a 72-h incubation at 37°C using each cell line.

### SP10 suppressed tumor growth in xenograft models by inducing apoptosis

**Figure 5:**
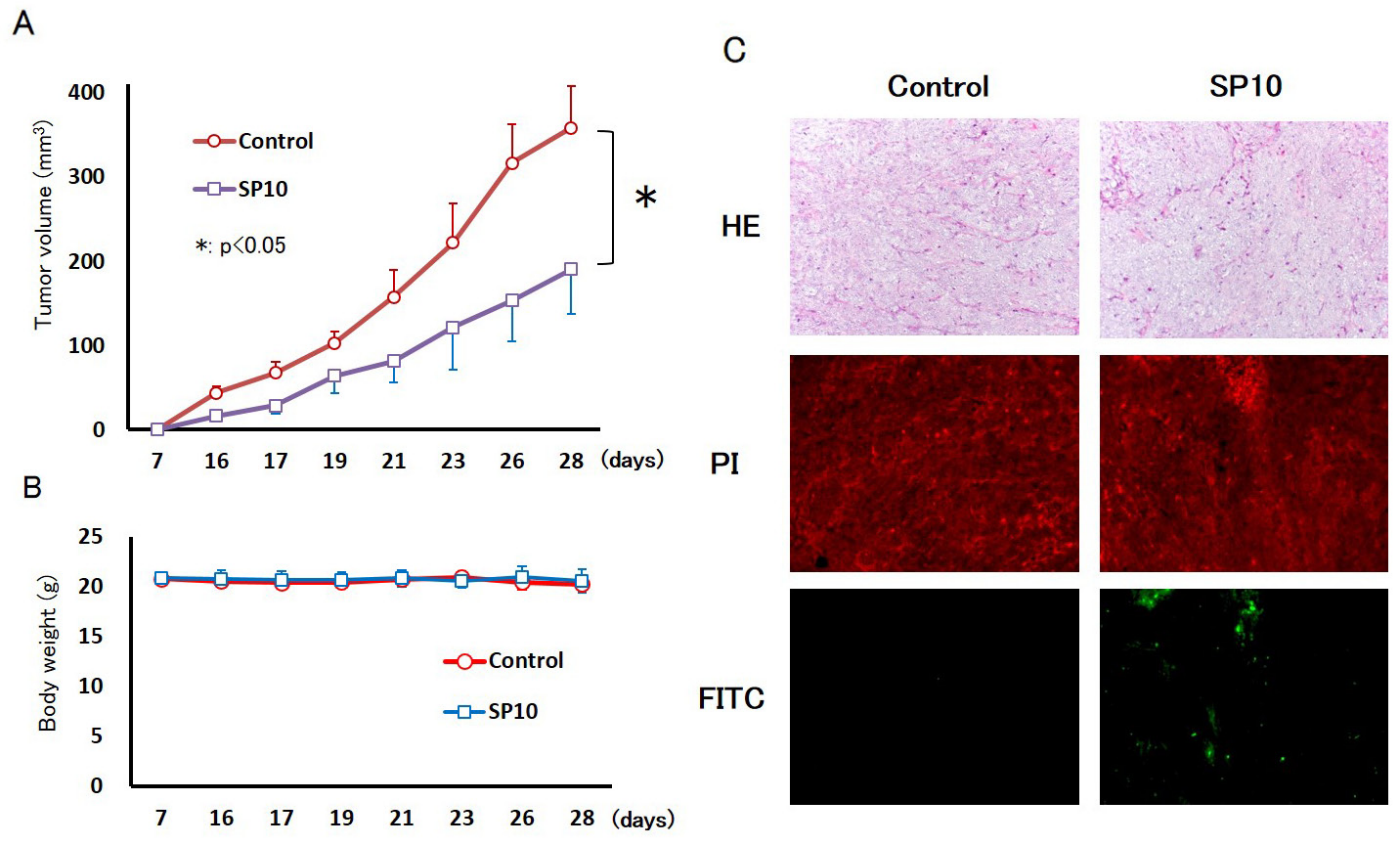
Anti-tumor effects of SP10 in an HCT116 tumor xenograft model **(A)** HCT116 cells (3×10^6^ per animal) were implanted subcutaneously into the right back flank of mice, and treatment commenced 7 days after tumor injection. SP10 (200 mg/kg orally, given 5 days/week for 21 days) effectively inhibited the growth of HCT116 allografts *in vivo* (^*^p<0.05). **(B)** Body weight did not change during the experiment. **(C)** TUNEL staining revealed apoptotic cells in the tumor tissue of the SP10 treatment group. Hematoxylin and eosin (H&E) staining was conducted of both the nuclei and cytoplasm. PI-stained nuclei were red in color. Apoptotic cells detected as FITC-positive cells were green color. Apoptotic cells were detected in SP10-treated tissue.

We expected SP10 to have stronger anti-tumor effects compared to SP6. Thus, to determine the effects of SP10 on tumor growth, we established an HCT116 tumor xenograft model using BALB/c nude mice. SP10 was orally administered at 200 mg/kg for 5 days/week. After 21 days of oral administration with vehicle control (distilled water), the tumor xenografts reached a mean volume of 358.6 ± 49.9 mm^3^. However, SP10 significantly decreased tumor growth (190.5 ± 53.0 mm^3^; Figure 5A). The body weight of mice did not change in both groups during the experiment (Figure 5B). To determine the mechanism underlying tumor suppression, tumors were collected and then TUNEL staining was performed, revealing apoptotic cells in the SP10 treatment group (Figure 5C). The percentage of apoptotic cells increased in the SP10 treatment group (Supplementary Figure 2B). Prussian blue staining was performed to detect ferric iron in the tumor tissue, and revealed positive blue spots that were only recognized in the stroma of the control group (Supplementary Figure 5). These results showed that SP10 suppressed tumor growth by inducing apoptosis, similar to the *in vitro* data.

### Acute oral toxicity and intravenous injection tests demonstrated the safety of SP6 and SP10

**Figure 6:**
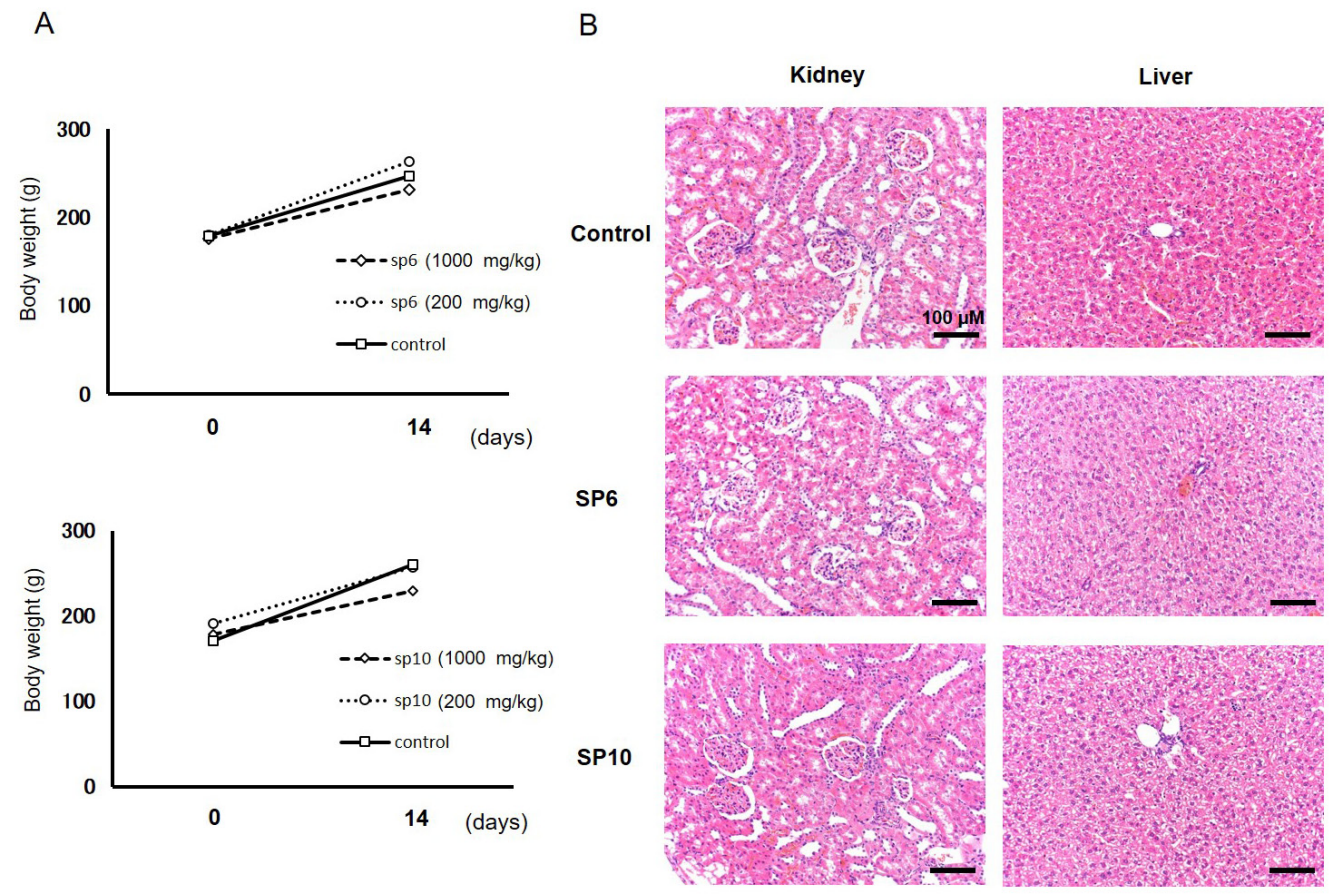
Evaluation of acute toxicity of SP6 and SP10 in rats **(A)** SP6 and SP10 were orally administered for 14 consecutive days. Body weight was not affected by SP6 and SP10 administration. **(B)** Kidney and liver organs were collected after examination, and H&E staining was performed. No disorder was detected in the SP6 and SP10 treatment groups.

Acute toxicity tests in rats were performed to evaluate the basic toxicity of SP6 and SP10. The agents were orally administered (600 and 1000 mg/kg), and the body weight of the rats did not change compared to the control group during the examination (Figure 6A). No abnormal behaviors were observed through the period of examination (Supplementary Table 1). A blood test was also performed in rats treated with SP6 (1000 mg/kg) and SP10 (1000 mg/kg), and no significant abnormalities were observed (Supplementary Table 2). To evaluate any possible adverse effects to the organs, we examined the liver and kidney tissues and found no injuries in the specimens (Figure 6B). To compare the safety of SP6 and SP10 to the known iron chelator DFO, an intravenous injection test was performed with all three agents. Although all mice died immediately after intravenous administration of DFO, none died after administration of SP6 or SP10 (Table 2). These results demonstrated that SP6 and SP10 are basically safe, and as such, are more beneficial for cancer therapy than DFO.

**Table 2:** Safety of intravenous injection of iron chelators in mice

Iron chelator		Vital result
DFO 300 mg/kg	1	Dead
	2	Dead
	3	Dead
	4	Dead
SP6 300 mg/kg	1	Alive
	2	Alive
	3	Alive
SP10 300 mg/kg	1	Alive
	2	Alive
	3	Alive

Each iron chelator was administered via the tail vein of Jcl:ICR mice. All mice injected with DFO died within 5 min, whereas those injected with SP6 or SP10 remained alive until they were sacrificed 2 weeks later.

## DISCUSSION

This is the first report of the efficacy and safety of SP for cancer therapy. Iron chelators were originally used to treat iron overload disease caused by hemochromatosis and blood transfusion [15, 16]. However, iron chelators have recently garnered attention as novel therapeutic agents for cancer, in part because their effectiveness was demonstrated in patients with sorafenib-refractory advanced hepatocellular carcinoma [17]. In addition, iron-rich food has been linked to an increased risk of carcinogenesis, especially breast cancer [18–21]. Thus, iron chelation therapy is expected to be a novel strategy for cancer therapy and prevention, particularly oral iron chelators compared to intravenous iron chelators due to their practicability. However, iron chelators cause potentially severe side effects, which makes them difficult to use in some cancer patients [22]. One study reported that all six hepatocellular carcinoma patients treated with DFX had side effects that led to rhabdomyolysis (increased creatinine kinase activity) or anorexia. Thus, decreasing the side effects of iron chelators may improve cancer treatment compliance, thereby improving clinical outcomes.

In this study, an acute toxicity test and blood test revealed that a high dose (1000 mg/kg) of SP6 and SP10 did not induce an abnormal appearance, cause a change in body weight, or cause organ dysfunction. A venous injection test revealed that SP6 and SP10 had a better safety profile than DFO. Although the oral iron chelator DFX was also suitable for comparison, our purchased DFX was not dissolved in distilled water and thus was technically difficult to use in the venous injection test. Our preliminary dose escalation intravenous injection test revealed that some mice were ill-conditioned with administration of over 500 mg/kg SP6 and SP10 (data not shown). Together, the results of this study showed that SP6 and SP10 are basically safe, and as such, are advantageous compared to DFO. SP was developed to have decreased side effects by avoiding metabolism via cytochrome P450, as drug metabolism via the cytochrome P450 system can cause drug interactions that result in drug toxicity. Because SP6 and SP10 are water soluble, it is thought to be unaffected by cytochrome P450. Although our blood and histological examinations did not show liver disease, the pharmacological mechanisms of SP are still unknown. There were fewer anti-proliferative effects of SP6 and SP10 in WI-38 and NIH-3T3 fibroblasts compared to the cancer cells (Supplementary Figure 1). A limitation of our study was the lack of more comprehensive toxicity tests. Additional studies are needed to confirm the safety of SP for clinical use for cancer therapy.

Iron chelation ability was assessed by the sulfosalicylic acid visual colorimetric method, which showed that the chelation ability of SP was stronger than that of sulfosalicylic acid. Moreover, SP10 had strong chelation ability compared to SP6, which was thought to induce apoptosis in this study. The induction of apoptosis by SP6 and SP10 was similar to previous reports of other iron chelators [7, 23, 24]. Although MCF-7 cells are caspase 3-deficient, caspase 3 is known to induce apoptosis with upregulation of cleaved PARP [14, 25]. We examined whether SP-6 and SP-10 were the trigger of apoptosis by using caspase-3 inhibitor. Induction of cleaved PARP was inhibited by caspase-3 inhibitor in HCS-2 cells, which indicated that SP-6 and SP10 triggered apoptosis (Supplementary Figure 2C). Induction of apoptosis was demonstrated in our *in vitro* and *in vivo* studies, which indicates that apoptosis may be the major anti-cancer mechanism of SP. The anti-tumor effects of SP6 were also confirmed in an HCT116 tumor xenograft model (Supplementary Figure 6A). Body weight did not change during the experiment (Supplementary Figure 6B). SP10 inhibited HCT116 tumor growth; however, the tumors did not disappear. Thus, further studies of these novel iron chelators combined with other anti-cancer agents should be performed to determine if their synergistic effects are more potent than treatment with either agent alone. We also examined the expression of iron-related proteins and ferrous ion (Fe^2+^) distribution in the cell (Supplementary Figures 3 and 4). Their expression tended to decrease, and Fe^2+^ distribution was condensed around the nuclei by administration of SP6 and SP10. These findings were evidence of iron chelation in the cancer cells.

In conclusion, SP is a novel oral iron chelator with anti-cancer effects via induction of apoptosis and few adverse side effects, and as such, may serve as a nontoxic iron chelator with high functionality. Iron chelators such as SP are expected to have anti-cancer effects not only by inhibiting cell proliferation via apoptosis but also by targeting cancer stem cells [26, 27].

## MATERIALS AND METHODS

### Cell culture

The human colon cancer cell line (HCT116) and breast cancer cell line (MCF-7) were purchased from American Type Culture Collection (Manassas, VA, USA). The human oral squamous cell carcinoma cell line (HSC-2), lung cancer cell line (A549), human fibroblast cell line (WI-38) and murine fibroblast cell line (NIH-3T3) were purchased from the National Institutes of Biomedical Innovation, Health and Nutrition (JCRB, Osaka, Japan). HSC-2, A549, MCF-7, WI-38, and NIH-3T3 cells were cultured in Dulbecco’s Modified Eagle’s Medium (Sigma-Aldrich, St. Louis, MO, USA) and HCT116 cells were cultured in RPMI-1640 medium (Sigma) at 37°C in humidified air with 5% CO_2_. The media were supplemented with 10% fetal calf serum (FCS) (Thermo Fisher Scientific, Waltham, MA, USA), 100 U/mL penicillin, and 100 μg/mL streptomycin (Sigma).

### Iron chelators

SP was provided by Disease Adsorption System Technologies Co., Ltd. (Kanazawa, Japan). It was chemically synthesized from catechol and benzoic acids. SP6 was made by a condensation reaction with glucosamine, and SP10 was made by a condensation reaction with histidine. SP was developed to have few side effects by avoiding metabolism by cytochrome P450. DFO was purchased from Novartis Pharma (Tokyo, Japan). SP6, SP10, and DFO were dissolved in distilled water at the indicated concentrations for *in vitro* and *in vivo* experiments.

### Chelation ability test

Fe^3+^ iron standard stock solution (Wako Pure Chemical Industries, Osaka, Japan) was used in this study. Fe^3+^ concentration was assessed by the sulfosalicylic acid visual colorimetric method using a kit (WAK-Fe^3+^, Kyoritsu chemical-check; Tokyo, Japan).

### Cell proliferation assay

The XTT cell proliferation assay (Cell Proliferation Kit II; Roche, Mannheim, Germany) was used to assess cell proliferation. The cells were seeded in medium plus 10% FCS. The cells were incubated with SP6 and SP10 for 48 h at 37°C in medium plus 1% FCS, and then changed to medium plus 1% FCS with SP6 and SP10 for 24 h.

Optical densities were measured at 480 and 650 nm. All experiments were performed in 96-well plates, and each experiment was repeated at least three times. The IC_50_ was calculated using Prism Windows software (version 6; GraphPad, La Jolla, CA, USA).

### TUNEL staining

The induction of apoptosis was assessed by the TUNEL assay using the MK500 in situ Apoptosis Detection Kit (Takara Bio, Shiga, Japan) according to the manufacturer’s protocol. Cancer cells were stained after treatment with SP6 and SP10 for 72 h. Living cells were fixed in 4% paraformaldehyde at 4°C for 30 min, and were treated with 70% ethanol to enhance permeability. Tumor tissue was also stained using the kit. The numbers of PI-positive and FITC-positive cells were calculated by Image J software (http://rsb.info.nih.gov/ij/).

### Ferrous ion staining

Fe^2+^ distribution was assessed by FeRhonox-1 staining kit (Goryo Chemical, Sapporo, Japan). Cancer cells were stained according to the manufacturer’s protocol after treatment with SP6 and SP10 for 72 h. Nuclei were counterstained with Hoechst nuclear stain and observed using the fluorescence BZ-X700 microscope (KEYENCE, Osaka, Japan).

### Western blotting

Proteins were extracted from whole cells after a 72 h incubation with medium and SP after the XTT assay was conducted. The concentrations of extracted protein were measured using standard protocols. Cells were lysed in cell lysis buffer (Cell Signaling Technology [CST], Danvers, MA, USA). Equal amounts of total cellular protein (18–30 μg/lane) were separated by sodium dodecyl sulfate polyacrylamide gel electrophoresis and electrophoretically transferred to polyvinylidene difluoride filter membranes (Bio-Rad, Hercules, CA, USA) according to the manufacturer’s protocol. The following primary antibodies were used: rabbit polyclonal anti-PARP antibody (No. 9542; CST), rabbit monoclonal anti-cleaved PARP antibody (No. 5625; CST), rabbit polyclonal anti-caspase 3 antibody (No. 9662; CST), rabbit monoclonal anti-cleaved caspase 3 antibody (No. 9664; CST), and rabbit monoclonal anti-GAPDH antibody (No. 5174; CST). All primary antibodies were used according to the manufacturer’s recommended dilution. All secondary antibodies (Santa Cruz Biotechnology, Dallas, TX, USA) were used at a 1:1000 dilution. The membranes were incubated with primary antibodies overnight at 4°C, followed by incubation with secondary antibodies. Chemi-Lumi One L (Nacalai, Kyoto, Japan) and Immuno Star LD (Wako Pure Chemical Industries) were used to detect the peroxidase activity of the secondary antibodies. A caspase-3 inhibitor was used to confirm the mechanism of apoptosis (235420-1MGCN; Merk, Darmstadt, Germany).

### Tumor allograft model

All animal experiments were performed according to the Japanese Welfare and Management of Animals Act, and were conducted in accordance with institutional guidelines at Shigei Medical Research Institute (Okayama, Japan). All animal experiments were approved by the Ethics Review Committee for Animal Experimentation of Shigei Medical Research Institute (Nos. 1709, 160401-1, and 160401-7). Female BALB/c (nu/nu) mice were purchased from CLEA Japan Inc. (Tokyo, Japan) and housed in sterile conditions. Experiments started when the mice were 8 weeks of age. HCT116 tumor cells in culture were harvested and re-suspended in a 1:1 ratio of phosphate-buffered saline and Matrigel (BD Biosciences, San Jose, CA, USA). Viable HCT116 cells (3.0×10^6^) were injected into the right back flanks of the mice subcutaneously. Tumor size and body weight were measured for 21 days. SP10 (200 mg/kg) was suspended in distilled water and administered orally using a probe 5 days/week. The treatment was started 7 days after inoculation (n=5). The control group received distilled water that was administered by local injection 5 days/week. The tumor volume was calculated by the following formula: Length × Width × Height × 0.52. This experiment was repeated after oral administration of 200 mg/kg SP6. At the end of the experiment, the animals were sacrificed and the tumors were collected for histological analysis. Paraffin sections were prepared from 10% formalin-fixed tumors and stained with hematoxylin and eosin and Prussian blue. Prussian blue staining was performed by incubating the fixed tissue in a mixture of 2% potassium ferrocyanide and 1% hydrogen chloride for 30 min.

### Acute oral toxicity test

A total of nine female rats weighing 166–186 g were used. Jcl:SD rats were purchased from CLEA Japan Inc. An acute toxicity test was performed following OECD Test Guideline 423. SP6 and SP10 were orally administered at 200 and 1000 mg/kg body weight. Rats were observed for toxic signs for 14 days, after which biochemical analysis of the collected blood was performed. The kidney and liver were collected for histopathological examination, and pathological evaluation was performed by pathologists.

### Venous injection test

A total of 10 male Jcl:ICR mice were used. DFO (300 mg/kg), SP6 (300 mg/kg), and SP10 (300 mg/kg) were administered via the tail vein. Mice were observed for survival for 14 days.

### Statistical analysis

Data were compared against the respective control in each experiment using Student’s *t*-test. P values less than 0.05 were considered statistically significant.

## SUPPLEMENTARY MATERIALS FIGURES AND TABLES



## Abbreviations

SP: super-polyphenol
DFX: deferasirox
DFO: deferoxamine
XTT: 2,3-Bis-(2-Methoxy-4-Nitro-5-Sulfophenyl)-2H-Tetrazolium-5-Carboxanilide
TUNEL: TdT-mediated dUTP nick end labeling
PARP: poly (ADP-ribose) polymerase
GAPDH: Glyceraldehyde-3-phosphate dehydrogenase
PI: Propidium iodide
FITC: Fluorescein isothiocyanate
H&E: Hematoxylin and Eosin
