# Supplementary material for: A novel, nontoxic iron chelator, super-polyphenol, effectively induces apoptosis in human cancer cell lines
Source: Oncotarget. 2018 Aug 28;9(67):32751–60. doi: 10.18632/oncotarget.25973 (PMC6132348; doi:10.18632/oncotarget.25973)
Supplement: Supplementary file 1 [file oncotarget-09-32751-s001.pdf]

A novel, nontoxic iron chelator, super-polyphenol, effectively induces apoptosis in human cancer cell lines

SUPPLEMENTARY MATERIALS

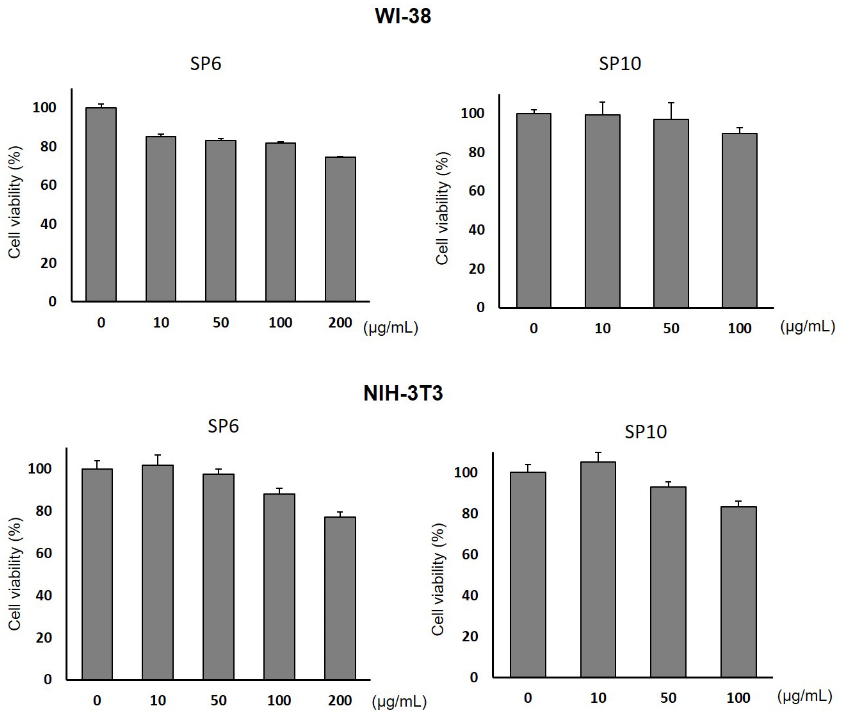

Supplementary Figure 1: Inhibitory effects of SP6 and SP10 in fibroblasts *in vitro*. WI-38 and NIH-3T3 cells were treated with different concentrations of SP6 and SP10 for 72 h, after which cell viability was evaluated using the XTT assay. Cell viability in the absence of treatment was set at 100%. The results are the means of three independent experiments. Data are presented as the mean ± SEM (n=4; *p<0.05).

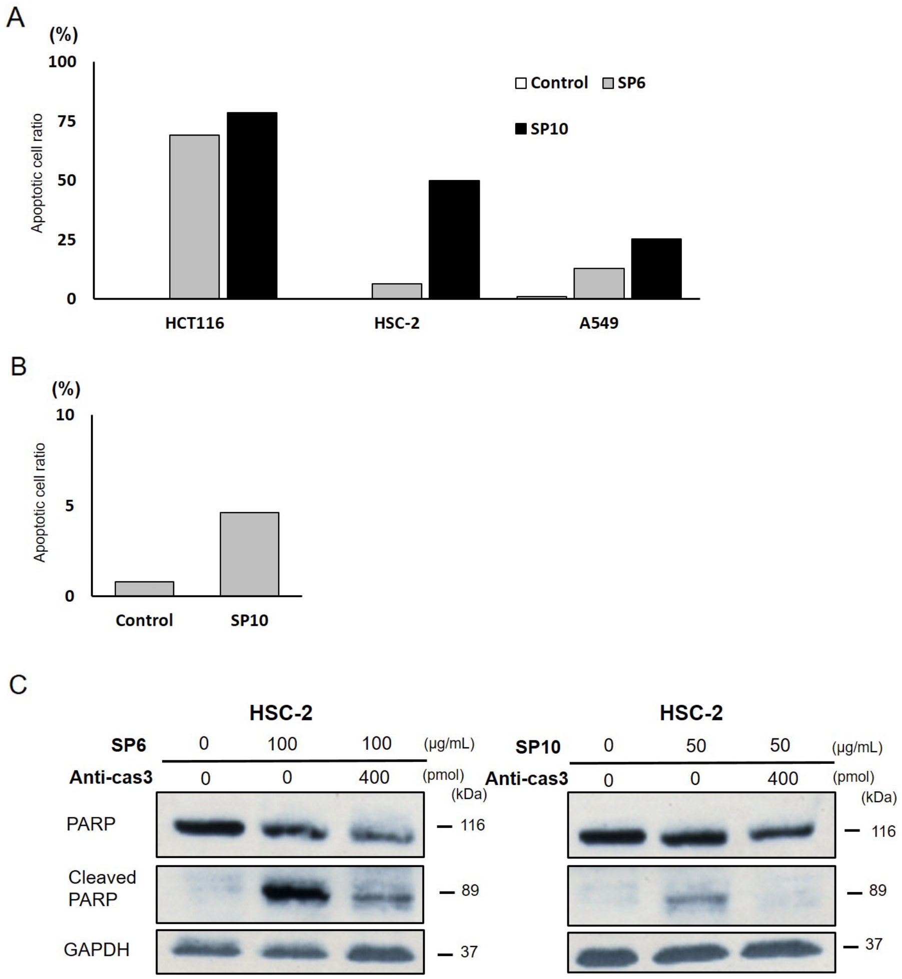

Supplementary Figure 2: Apoptotic effects of SP6 and SP10 in *in vitro* and *in vivo*. **(A)** PI-positive cells and FITC-positive cells in Figure 3 were counted by Image J software. The TUNEL staining positive cell ratio was calculated as FITC-positive cells/PI-positive cells. **(B)** The PI-positive cells and FITC-positive cells in Figure 5C were counted by Image J software. The TUNEL staining positive cell ratio was calculated as FITC-positive cells/PI-positive cells. **(C)** Cultured HSC-2 cells were treated with SP6 (100 μg/mL), SP10 (50 μg/mL), and caspase-3 inhibitor for 72 h. Then the cells were harvested and expression of the indicated proteins was analyzed. The caspase 3 inhibitor inhibited the induction of apoptosis via PARP cleavage.

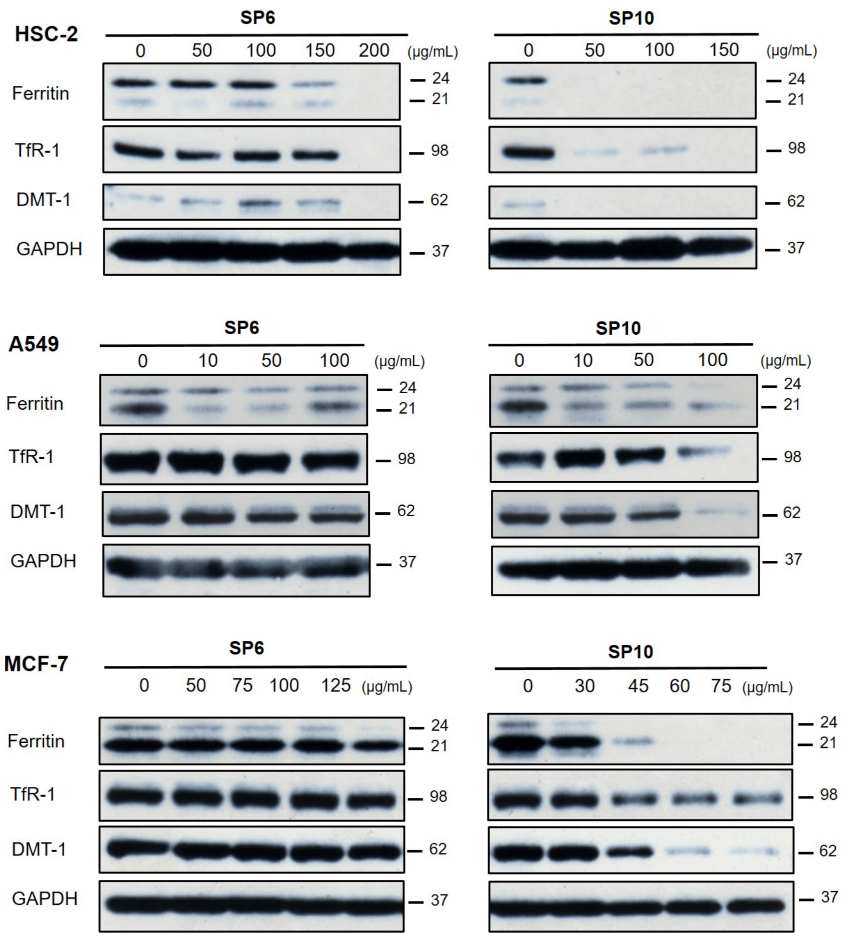

Supplementary Figure 3: Western blot analysis of iron-related proteins *in vitro*. HSC-2, A549, and MCF-7 cells were treated with different concentrations of SP6 and SP10 for 72 h. Then the cells were harvested and expression of the indicated proteins was analyzed. The expression of iron-related proteins tended to decrease by SP6 and SP10 administration.

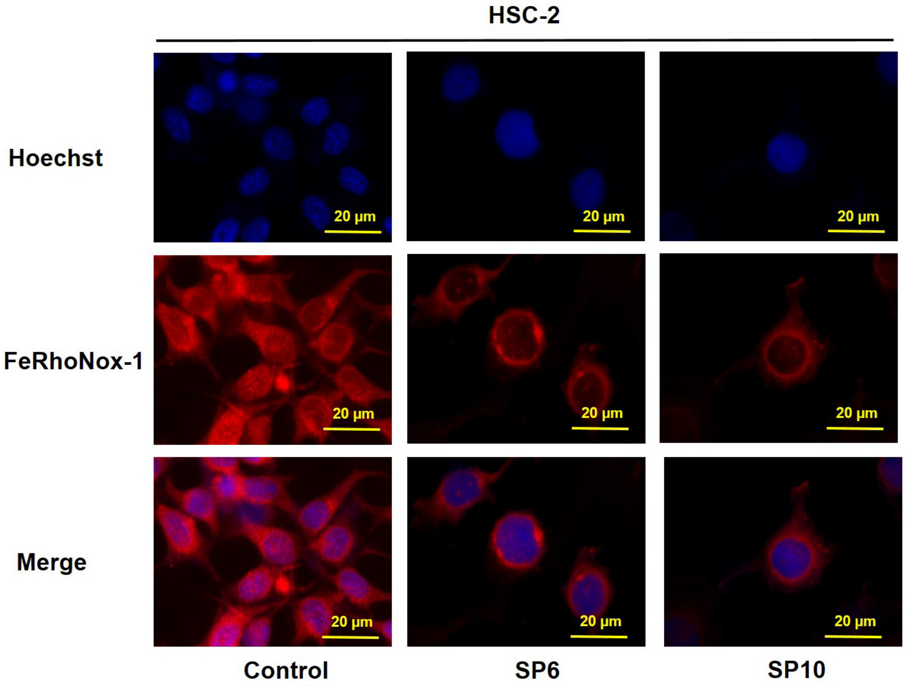

Supplementary Figure 4: Ferrous ion staining *in vitro*. Cultured HSC-2 cells were treated with SP6 (100 µg/mL) and SP10 (50 µg/mL) for 72 h, FeRhonox-1 staining was performed to detect Fe^{2+}. Hoechst staining revealed the nuclei and FeRhonox-1 staining revealed Fe^{2+}. The distribution of Ferrous was condensed around the nuclei by SP6 and SP10 administration.

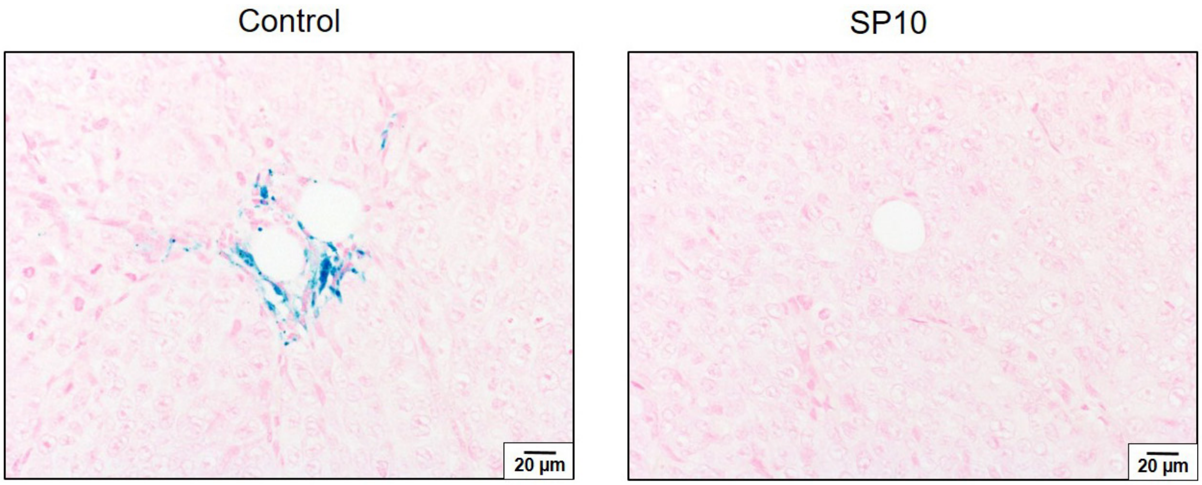

Supplementary Figure 5: Prussian blue staining of SP10 in an HCT116 tumor xenograft model. Resected tumors were analyzed for Fe^{3+} by Prussian blue staining. Positive blue spots were not detected in the SP10 treatment group.

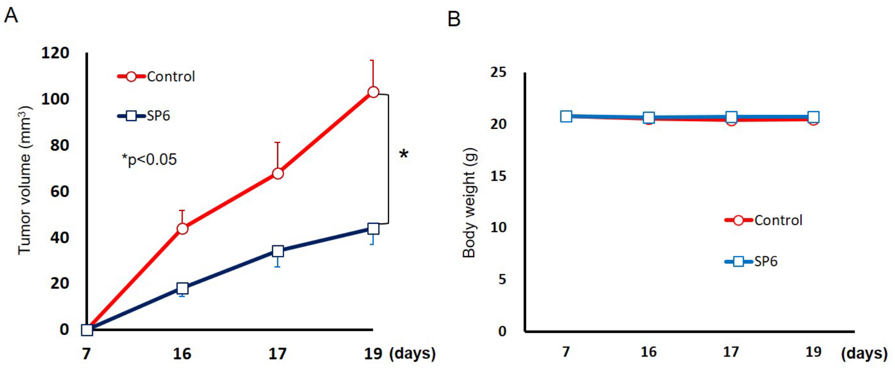

Supplementary Figure 6: Anti-tumor effects of SP6 in an HCT116 tumor xenograft model. **(A)** HCT116 cells (3×10^6 per animal) were implanted subcutaneously into the right back flank of the mice, and treatment commenced 7 days after tumor injection. SP6 (200 mg/kg orally, given 5 days/week) effectively inhibited the growth of HCT116 allografts *in vivo* ($^*p<0.05$). **(B)** Body weight did not change during the experiment.

Supplementary Table 1: Assessment of acute toxicity of SP6 and SP10, as determined by effects on appearance

			**Control**						**SP6**						**SP10**					
**Days**			**0**			**14**			**0**			**14**			**0**			**14**		
**Observation item**			**1**	**2**	**3**	**1**	**2**	**3**	**1**	**2**	**3**	**1**	**2**	**3**	**1**	**2**	**3**	**1**	**2**	**3**
Through cage		External appearance	○	○	○	○	○	○	○	○	○	○	○	○	○	○	○	○	○	○
		Behavior	○	○	○	○	○	○	○	○	○	○	○	○	○	○	○	○	○	○
		Spasm	○	○	○	○	○	○	○	○	○	○	○	○	○	○	○	○	○	○
		Respiration	○	○	○	○	○	○	○	○	○	○	○	○	○	○	○	○	○	○
		Position	○	○	○	○	○	○	○	○	○	○	○	○	○	○	○	○	○	○
		Sound reflection	○	○	○	○	○	○	○	○	○	○	○	○	○	○	○	○	○	○
Handling	Holding tail	Abdomen	○	○	○	○	○	○	○	○	○	○	○	○	○	○	○	○	○	○
	Holding body	Reflection	○	○	○	○	○	○	○	○	○	○	○	○	○	○	○	○	○	○
		Eye, Nose, Mouse	○	○	○	○	○	○	○	○	○	○	○	○	○	○	○	○	○	○
		Skin	○	○	○	○	○	○	○	○	○	○	○	○	○	○	○	○	○	○
		Thoracicoabdomen	○	○	○	○	○	○	○	○	○	○	○	○	○	○	○	○	○	○
		Musculus tonus	○	○	○	○	○	○	○	○	○	○	○	○	○	○	○	○	○	○
		Body temperature	○	○	○	○	○	○	○	○	○	○	○	○	○	○	○	○	○	○
		Breath sound	○	○	○	○	○	○	○	○	○	○	○	○	○	○	○	○	○	○
On cage		Behavior	○	○	○	○	○	○	○	○	○	○	○	○	○	○	○	○	○	○
		Head	○	○	○	○	○	○	○	○	○	○	○	○	○	○	○	○	○	○

The appearance of rats was recorded at the beginning and end of examination (n=3). Neither SP6 nor SP10 affected appearance.

Supplementary Table 2: Results of blood tests in rats after acute toxicity examination

	Control	SP6	SP10	
BUN	25.6±1.9	26.1±3.5	23.3±1.1	mg/dL
CRE	0.38±0.06	0.39±0.09	0.36±0.01	mg/dL
Na	144.7±0.6	142.7±1.5	143.7±2.1	mEQ/L
K	5.5±1.6	4.1±0.4	5.3±0.7	mEQ/L
Ca	11.1±0.4	10.5±0.5	10.8±0.4	mg/dL
TP	5.57±0.2	5.2±0.2	5.5±0.2	g/dL
T-Bil	0.06±0.02	0.03±0.02	0.04±0.03	mg/dL
AST	63.3±12.7	96.3±71.7	69.0±18.4	IU/L
ALT	28.3±8.7	51.7±36.8	27.7±4.6	IU/L
ALP	582.0±110.0	551.0±168.9	604.4±13.1	IU/L
γ-GTP	>3	>3	>3	IU/L

Blood tests were performed after 14 days of observation in the acute toxicity test (n=3).
